# The Role of SUMO-Conjugating Enzyme Ubc9 in the Neuroprotection of Isoflurane Preconditioning Against Ischemic Neuronal Injury

**DOI:** 10.1007/s12035-014-8797-3

**Published:** 2014-06-25

**Authors:** Li Tong, Zhixin Wu, Mingzi Ran, Yu Chen, Lujia Yang, Haopeng Zhang, Lina Zhang, Hailong Dong, Lize Xiong

**Affiliations:** 1 Department of Anesthesiology, Xijing Hospital, Xi’an, Shaanxi 710032 China; 2 Department of Anesthesiology, Chinese PLA General Hospital, Beijing, 100853 China

**Keywords:** Isoflurane, Ischemia, Ubc9, Neuroprotection, Preconditioning

## Abstract

Preconditioning with volatile anesthetics can create an ischemia tolerance against cerebral ischemia-reperfusion injury. We investigated whether ubiquitin conjugase 9 (Ubc9), the E2 conjugase for SUMOylation, is associated with neuroprotection induced by isoflurane preconditioning (IsoPC). In vitro, Ubc9 protein expression was evaluated at 4 and 24 h after reoxygenation. The role of Ubc9 in the neuroprotective effect was assessed in the presence or absence of Ubc9 small interfering RNA (siRNA). In vivo, rats were preconditionally exposed for 1 h to 2 % isoflurane for five consecutive days followed by middle cerebral artery occlusion. Neurobehavioral scores and infarction volume were determined at different times after reperfusion. The role of Ubc9 in ischemic tolerance was evaluated by intracerebroventricular microinjection with the Ubc9 siRNA. We showed that isoflurane preconditioning improved the cell viability of the SH-SY5Y cells that were challenged by oxygen-glucose deprivation. It also reduced brain infarct volumes and improved neurologic outcomes in the focal cerebral ischemic rat. The expression of Ubc9 was upregulated by isoflurane preconditioning. Knockdown of Ubc9 significantly attenuated the isoflurane preconditioning-induced neuroprotective effects. Isoflurane preconditioning-induced neuroprotection against ischemic injuries is mediated by Ubc9. These results suggest a novel mechanism for isoflurane preconditioning-induced tolerance to cerebral ischemia.

## Introduction

Perioperative stroke is one of the most significant complications of cardio-cerebral surgery, since it causes high mortality and severe disability [1]. Ischemic preconditioning is a technique that activates cellular pathways and can help induce tolerance to severe ischemia insults in the heart and brain [2]. Recently, our group and others have shown that exposing rats to inhaled anesthetics, such as isoflurane or sevoflurane, prior to ischemic insults can induce a tolerance to subsequent ischemia in the brain [3], spinal cord [4], and heart [5]. As volatile anesthetic agents have been proven safe in clinical treatments, they may be applied as a more medically feasible approach for preventing ischemic insults in the future. However, the exact molecular and cellular mechanisms underlying the protective effects of preconditioning remain unclear.

In 2004, a novel protein degradation pathway, ubiquitination, was found to have a crucial role in protein modulation [6]. The ubiquitin pathway is the major non-lysosomal system for intracellular protein degradation. The small ubiquitin-related modifier (SUMO) modification pathway is similar to ubiquitin, and SUMOylation is modulated by an enzymatic cascade analogous to that involved in ubiquitination [7]. SUMO proteins act by covalent attachment to lysine residues on larger proteins [8]. While several SUMO enzymatic cascades that are specific to the respective target proteins have been identified, ubiquitin conjugase 9 (Ubc9) is only SUMO-E2-conjugating enzyme. Therefore, Ubc9 is an ideal target for activation or blockage of the SUMO modification pathway. Isopeptidases catalyze de-SUMOylation and thus balance Ubc9 conjugation activities to modulate steady state levels of SUMO conjugates [9, 10].

Recent evidence has shown that the target protein SUMOylation has an important neuroprotective role in the cellular response to cerebral ischemia injury. It was first identified that SUMO1- and SUMO2/SUMO3-targeted modification of cellular proteins occurs in the ground squirrel during hibernating torpor, which suggests that SUMOylation may also be involved in hibernation-mediated neuroprotective effects [11]. Other researchers have shown that global SUMOylation, a posttranslational protein modification, occurs during ischemic tolerance in cultured cortical neurons and animal brains [2, 12, 13]. Lee et al. showed that Ubc9 transgenic mice have elevated levels of SUMO conjugation in the brain and increased resistance to brain ischemia compared to wild-type mice [14]. It is therefore of key clinical interest to identify the consequences of protein SUMOylation after preconditioning and ischemia to better understand the significance of this process in the fate of postischemic cells and animals. Our previous study found that preconditioning with isoflurane significantly alleviates cerebral ischemic injury by inhibiting ubiquitin-conjugated protein accumulation following mouse models of global cerebral ischemia. However, whether SUMOylation is associated with the neuroprotective mechanisms induced by isoflurane preconditioning remains unclear. Since evidence has suggested that the level of Ubc9 protein was positively correlated with the degree of SUMOylation, we hypothesized that Ubc9 (the sole conjugase for SUMOs) target proteins have a role in the mediation of the neuroprotective effect against cerebral ischemia afforded by isoflurane preconditioning. We tested this hypothesis using both in vitro and in vivo experimental settings.

## Materials and Methods

### Animals and Cells

All procedures were carried out according to the protocols approved by the Ethics Committee for Animal Experimentation of the Fourth Military Medical University (Xi’an, China) and in accordance with the National Institutes of Health Guide for the Care and Use of Laboratory Animals. SH-SY5Y cells used in the in vitro experiments were purchased from the American Type Culture Collection (ATCC, Manassas, VA, USA). Adult male Sprague–Dawley rats (280–320 g) were purchased from the Laboratory Animal Center of the Fourth Military Medicine University.

### Experimental Procedures

#### In Vitro

##### Experiment 1: Identification of the Alteration of Ubc9 Expression in Differentiated SH-SY5Y Cells After Oxygen-Glucose Deprivation (OGD) and Isoflurane Preconditioning

Human SH-SY5Y neuroblastoma cells were stimulated using all-trans retinoic acid (RA) in culture for several days to allow them to differentiate into neuron-like cells. They were then divided into four treatment groups: sham, Iso (isoflurane alone), OGD, and IsoPC (isoflurane preconditioning + OGD) groups. At 24 h after OGD, cell viability was evaluated. The cells were harvested at 4 and 24 h after reoxygenation to measure the expression of Ubc9 protein using Western blotting and immunofluorescence.

##### Experiment 2: Verification of the Role of Ubc9 in Mediating the Neuroprotective Effect Induced by Isoflurane Preconditioning Against OGD in Differentiated SH-SY5Y Cells

Small interference RNA (siRNA) and lentivirus vectors were used. Cells were assigned to eight groups: sham, Iso, OGD, IsoPC (isoflurane preconditioning + OGD), siRNA + OGD, siRNA + IsoPC (siRNA + isoflurane preconditioning + OGD), lenti + OGD (lentivirus vector + OGD), and lenti + IsoPC (lentivirus vector + isoflurane preconditioning + OGD). The MTT assay was used 24 h after reoxygenation to measure the viabilities of the neuron-like cells.

#### In Vivo

##### Experiment 1: Examination of Ubc9 Expression Following Focal Cerebral Ischemia and Isoflurane Preconditioning in the Rat

Rats were randomly allocated to three groups (*n* = 8 each): sham, middle cerebral artery occlusion (MCAO), and IsoPC (isoflurane preconditioning + MCAO). At 24 h, 48 h, 72 h, 7 days, and 14 days after reperfusion, functional neurologic outcomes were evaluated. Cerebral infarct volume was observed at 72 h, 7 days, and 14 days. The other six rats from each group were killed 4 h after reperfusion to evaluate Ubc9 protein expression.

##### Experiment 2: Verification of the Role of Ubc9 in Mediating the Neuroprotective Effect Induced by Isoflurane Against Focal Cerebral Ischemia in Rats

We constructed a siRNA against Ubc9. The reliability of the siRNA was tested in normal rats before use. To investigate the impact of siRNA on the neuroprotective effect of isoflurane pretreatment, the rats were randomly divided into five groups: sham, MCAO, IsoPC (isoflurane preconditioning + MCAO), siRNA + IsoPC (Ubc9 siRNA + isoflurane preconditioning + MCAO), and siRNA-c + IsoPC (control siRNA + isoflurane preconditioning + MCAO) groups. The effect of siRNA on functional neurologic outcomes was evaluated at 24, 48, and 72 h after ischemia. Cerebral infarct volume was compared at 72 h after ischemia-reperfusion among the different groups.

#### Cell Culture and Characterization

The human neuroblastoma cell line, SH-SY5Y, is often induced by stimulation with RA to differentiate into neuron-like cells and has been used to imitate the responses of neurons [15]. In the present study, exponentially growing SH-SY5Y cells were kept in airtight, temperature-controlled cell culture chambers built specifically for these experiments. Cells were maintained in a mixture containing 1:1 Ham’s F12 and Dulbecco’s modified Eagle’s medium (DMEM) supplemented with 10 % heat-inactivated fetal bovine serum, 2 mM glutamine, 0.28 μg/μL of gentamicin, and 250 μg of amphotericin B in a humidified atmosphere of 5 % CO_2_ in air at 37 °C until 90 % confluence was reached. Then, 10 μM of RA (1 % of the total volume, R2625; Sigma, St Louis MO, USA) was added into the medium to stimulate the cells to differentiate. Culture medium was replaced once every 2 days.

#### In Vitro Isoflurane Preconditioning and OGD

In the in vitro study, preconditioning was achieved by incubating the cells in 2 % isoflurane with 98 % O_2_ for 2 h [16]. Cells were then returned to the incubator filled with air and 5 % CO_2_ and continuously cultured for 24 h. To induce ischemic injury, the neuron-like cells were subjected to OGD treatment. Cultures were changed with prewarmed OGD medium and then put into an airtight chamber at 37 °C for 3 h. The OGD medium contained 116 mM NaCl, 5.4 mM KCl, 0.8 mM MgSO_4_, 1.0 mM NaH_2_PO_4_, 1.8 mM CaCl_2_, and 26 mM NaHCO_3_ and was bubbled with 5 % CO_2_ and 95 % N_2_. OGD was terminated by removing cultures from the chamber and changing the media back to the normal culture medium. Cells were then cultured for an additional 2 h.

#### Assay of Cell Viability

Cell viability was determined using the MTT assay, a mitochondrial enzyme-dependent reaction. Briefly, MTT was added to the cells cultured in 96-well plates after completion of the experimental treatments. In metabolically active cells, the yellow tetrazolium MTT salt is cleaved into purple formazan crystals. Thus, formed formazan can be solubilized and the absorbance measured using a multiplate reader at 490 nm.

#### Analysis of Cell Apoptosis by Flow Cytometry

A Calibur Flow Cytometer system (BD Inc., USA) was used to analyze the cell cycle distribution and apoptosis. The SH-SY5Y cells used for the detection of cell cycle distribution were stained with propidium iodide (PI). A FITC Annexin V Apoptosis Detection Kit (BD Pharmingen, USA) was used to detect apoptosis.

#### Interference of Ubc9 on Cells

RNA interference experiments to suppress Ubc9 expression in cells were performed using the FlexiTube siRNA Premix (Qiagen, Hilden, Germany) and processed according to the manufacturer’s instructions. The following target sequence was used: 5′-ACCACCATTATTTCACCCGAA-3′. Neuroblastoma cell lines were cultured in a 6-well plate at a density of 3 × 10^5^ cells/well for 24 h and transfected with 5.5 μg siRNA using Lipofectamine^tm^. After 48 h, siRNA validation was determined using an assay for Ubc9 expression.

Viral particles were made in HEK-293 T cells (ATCC, cat. no. CRL-11268) by cotransfection of the lentiviral vector Ubc9 and the Packaging Plasmid Mix with Lipofectamine^tm^ 2000. The culture medium containing the packaged viruses was harvested 48 h after transfection and spun at 3,000 rpm for 20 min at 4 °C. The transduced cells were harvested after 72 h to check for RNA interference efficiency.

#### Transfection of siRNA into the Rat Brain

First, we tested the efficiency of the siRNA against Ubc9 in normal rats. The following target sequence was used: 5′-TTAGAGTAAATAAACTGTTTA-3′. We performed siRNA transfections in the rats according to the method described by Luo et al. [17].

After the efficiency of siRNA was verified, we assessed the impact of administration of siRNA Ubc9 on the protective effect of isoflurane preconditioning in the cerebral ischemic rat. Animals were randomly divided into five groups: sham (without intervention), siRNA (Ubc9 siRNA), siRNA-c (control siRNA), siRNA + IsoPC (Ubc9 siRNA followed by isoflurane preconditioning + MCAO), and siRNA-c + IsoPC (control siRNA followed by isoflurane preconditioning + MCAO).

Under pentobarbital anesthesia (40 mg/kg, i.p.), a stainless steel cannula was stereotaxically implanted into the unilateral cerebral ventricle. The stereotaxic coordinates of the lateral cerebral ventricle were 1.0 mm posterior to the bregma and 1.2–1.5 mm lateral to the midsagittal line [18]. In total, 5 μL of the diluted mixture was stereotaxically delivered into the ipsilateral lateral ventricle. After recovering from anesthesia, the rats were returned to their cages and allowed access to food and water ad libitum. The animals were subjected to subsequent treatments 24 h later.

#### Surgery for Transient Middle Cerebral Artery Occlusion

The transient MCAO model was performed as previously described [19]. After an overnight rest, animals were anesthetized using 2 % isoflurane [20]. After the neck skin was cut open and the carotid arteries were removed, transient focal cerebral ischemia was induced by intraluminal suture occlusion of the right middle cerebral artery with a 3-0 monofilament nylon suture (Ethicon, Inc., Osaka, Japan). After 120 min of MCAO, the suture was carefully removed from the internal carotid artery. Sham-operated rats underwent the same surgical procedure except that the suture was not inserted into the internal carotid artery. The cerebral blood flow (CBF) in parietal cortex was continuously monitored with laser Doppler flowmetry (PeriFlux 5000, Sweden). The rat was considered as a successful model when its CBF reduced to 30 % of the basal value.

#### Isoflurane Preconditioning In Vivo

For the in vivo study, preconditioning of the rats was achieved by inhalation of 2 % isoflurane + 98 % O_2_ 1 h/day for 5 days [21]. Animals in the control group were treated for the same duration but were allowed to inhale 98 % O_2_ only.

#### Measurement of Physiologic Parameters

As isoflurane inhalation may induce hypoxia, it was necessary to rule out hypoxia-induced preconditioning. Therefore, in a separate experiment, ten additional rats were used to measure blood gases during isoflurane preconditioning (*n* = 5) or oxygen inhalation (*n* = 5). A PE-10 cannula (Becton Dickinson and Company, Rutherford, NJ, USA) was inserted into the left femoral artery using a carrier amplifier connected to an invasive pressure monitor (Spacelabs Medical, Inc., Redmond, WA, USA). Arterial blood gas samples were collected at the beginning, during, and just before termination of preconditioning. Samples were then measured using an OMNI Modular System (AVL List GmbH Medizintechnik, Kleiststrasse, Graz, Austria).

Since the surgical procedure of the MCAO model was very short (average time for intraluminal suture insertion was 5 min and the removal time was 1–2 min), the physiological parameters were not measured during the surgical procedure according to previous reports [22]. However, a separate experiment was undertaken to measure the physiological parameter changes during isoflurane preconditioning. The results showed that no significant differences in physiological parameters were found between the sham and MCAO groups (data not shown).

#### Evaluation of Neurologic Scores and Measurement of Infarct Volume

Neurologic scores were evaluated by using a scoring system reported by Garcia et al. [23]. This system consisted of the following six tests: (1) spontaneous activity, (2) symmetry in the movement of four limbs, (3) forepaw outstretching, (4) climbing, (5) body proprioception, and (6) response to vibrissae touch. The final score given to each rat at the completion of the evaluation was the summation of all six individual test scores. The minimum neurologic score was 3 and the maximum score was 18.

To compare the infarct sizes among differently treated groups, the infarct volume was measured using 2,3,5-triphenyltetrazolium chloride (TTC) staining (Sigma-Aldrich, St Louis, MO, USA) as described in our previous report [21]. Infarct and total hemispheric sections were traced from slices taken at 2-mm intervals and measured using an image analysis system (Adobe Photoshop 8.0, Adobe Systems Incorporated, San Jose, CA, USA). The infarct volume was calculated using Swanson’s method to correct for edema: 100 × (contralateral hemisphere volume − non-lesioned ipsilateral hemisphere volume) / contralateral hemisphere volume [24] and presented as mean ± SEM.

#### Western Blot

In the first part of the experiment, to assess the expression of Ubc9 proteins, cells seeded in 75 cm^3^ bottles at a density of 5 × 10^6^ were washed with phosphate-buffered saline (PBS) and resuspended in a Tris buffer (pH 7.0) containing protease inhibitors (Roche, F. Hoffmann-La, Basel, Switzerland). After sonication, 20 μg of the total protein extracts were separated by sodium dodecyl sulfate-polyacrylamide gel electrophoresis (SDS-PAGE) and then transferred onto a PVDF membrane. Thereafter, non-specific binding was blocked with 5 % bovine serum albumin in TBST for 1 h at room temperature. Membranes were then incubated overnight at 4 °C with rabbit anti-Ubc9 (1:2000, ab6046; Abcam, Cambridge, UK) and rabbit anti-β-actin (1:4000, ab6046; Abcam), respectively. After rinsing, the membranes were incubated with an anti-rabbit secondary antibody for 2 h at room temperature. Bands were visualized using the SuperSignal West Pico Chemiluminescence Substrate from Pierce (Rockford, IL, USA).

In the second part of the experiment, to examine the expression of Ubc9, the animals were decapitated and the cerebral cortex was rapidly harvested. Subsequent protocols were the same as above.

#### Immunocytofluorescence Staining

In the in vitro experiment, at the end of treatment, cells were grown on 22-mm sterilized glass coverslips fixed with 4 % paraformaldehyde for 30 min and then thoroughly rinsed with PBS. Non-specific binding sites were blocked with 5 % (*v*/*v*) normal goat serum in PBS for 1 h at room temperature. The coverslips were then incubated with appropriate dilutions of primary antibodies in PBS for 24 h at 4 °C. The primary antibodies used were rabbit anti-Ubc9 (1:200). The sources of primary antibodies were the same as those used in the Western blotting procedures. Then, the coverslips were washed with PBS and incubated with suitable secondary antibodies conjugated with fluorescence for 2 h at room temperature. The cellular nuclei were counterstained with DAPI. The coverslips were then washed extensively and mounted on glass slides with anti-fading medium for image observation and analysis. Images were captured with a Nikon E600 fluorescent microscope (Nikon, Tokyo, Japan).

### Statistical Analysis

SPSS 13.0 for Windows was used to carry out statistical analyses. All values, except for neurological scores, are presented as mean ± SEM and were analyzed by one-way analysis of variance. The neurological scores were expressed as the median (range) and were analyzed with the Kruskal–Wallis test followed by the Mann–Whitney *U* test with Bonferroni correction. Values of *p* < 0.05 were considered statistically significant.

## Results

### Isoflurane Preconditioning Induces Neuroprotection Against OGD In Vitro

**Fig. 1:**
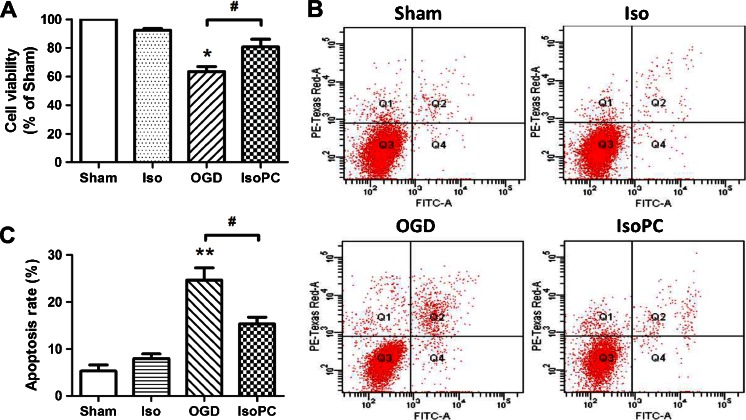
*a* Cell viabilities indicated by the MTT values in each experimental group. The values in *vertical axis* indicate the percentage of cells in each group compared to the sham (*n* = 6 for each group). Data are presented as mean ± SEM. *p < 0.05 compared with the sham group. #*p* < 0.05 compared with the OGD group. **b**, **c** Anti-apoptotic effects of isoflurane preconditioning in cells subjected to OGD as indicated using Annexin V-FITC double staining and flow cytometry

We first investigated the neuroprotective effects of isoflurane preconditioning in vitro by MTT assay and flow cytometry analysis. The results of cell viability and apoptosis revealed that isoflurane preconditioning alleviated differentiated SH-SY5Y cell death induced by 3 h of OGD injury (*p* < 0.05; Fig. 1a). Quantitative analysis of the neuron-like cells revealed that 2 h of isoflurane preconditioning given prior to OGD exposure significantly decreased cell apoptosis, compared with that in OGD injured alone (*p* < 0.05; Fig. 1b, c).

### Isoflurane Preconditioning Altered the Expression of Ubc9 In Vitro

**Fig. 2:**
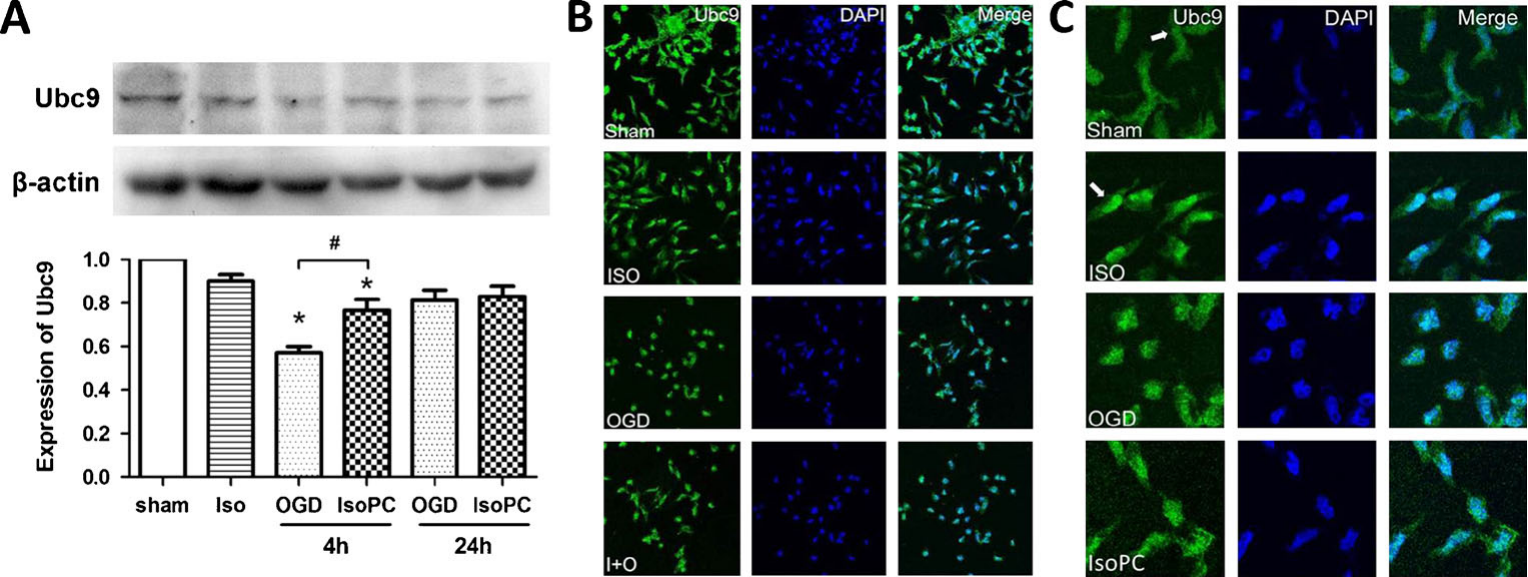
**a** Western blot results showing isoflurane preconditioning-induced recovery of Ubc9 expression hampered by OGD (*n* = 6 for each group). Data are presented as mean ± SEM. *p < 0.05 compared with the sham group. #*p* < 0.05 compared with the OGD 4-h group. **b**, **c** Representative immunofluorescent images for Ubc9 expression in different groups. Ubc9-positive cells were shown in *green* and cell nuclei were stained with DAPI (*blue*) (color figure online)

We investigated the effects of isoflurane preconditioning on the expression of Ubc9 (SUMO E2 conjugation enzyme) using Western blotting. The results showed that the incubation with OGD decreased the levels of Ubc9 protein in differentiated SH-SY5Y cells. The expression of Ubc9 levels was significantly reduced at 3 h after OGD (*p* < 0.05), but there was no statistical difference between the sham group and the 24-h OGD group. The reduction of Ubc9 protein expression induced by OGD was attenuated by isoflurane preconditioning (Fig. 2a). We also investigated the expression of Ubc9 protein 3 h after OGD using immunofluorescence, and these images showed the same results (Fig. 2b). Moreover, isoflurane preconditioning triggered nuclear translocation of the SUMO-conjugating enzyme Ubc9 (Fig. 2c).

### Manipulation of Ubc9 Levels Affects Isoflurane-Induced Neuroprotection

**Fig. 3:**
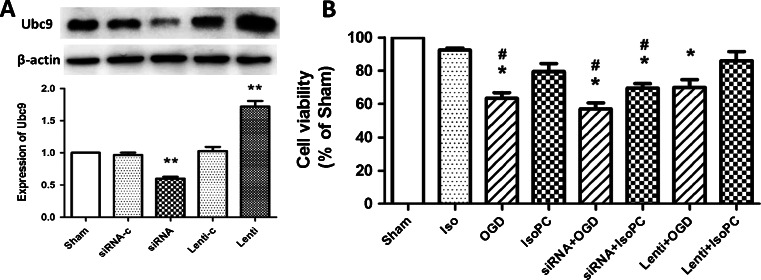
**a** Western blot analysis showed the efficiency of Ubc9-lentivirus and Ubc9 siRNA on the expression of Ubc9 in differentiated SH-SY5Y cells. Sham (cells without treatment); siRNA-c (cells treated with Allstars Negative Control siRNA); Ubc9 siRNA (cells treated with Ubc9 siRNA); lenti-c (cells treated with control lentivirus); Ubc9 lenti (cells treated with Ubc9 lentivirus). Data are presented as mean ± SEM. **p < 0.01 compared with the sham group. **b** Histograms showing the percentage of cell viabilities in Ubc9 siRNA or Ubc9 lentivirus-transfected cells and non-transfected cells when subjected to different treatments. Sham (without treatment), Iso (isoflurane only), OGD (oxygen-glucose deprivation), IsoPC (isoflurane preconditioning plus OGD), siRNA + OGD (Ubc9 siRNA plus OGD), siRNA + IsoPC (Ubc9 siRNA plus isoflurane preconditioning plus OGD), lenti + OGD (Ubc9 lentivirus plus OGD), lenti + IsoPC (Ubc-lentivirus plus isoflurane preconditioning plus OGD). Data are presented as mean ± SEM. *p < 0.05 compared with the sham group. #*p* < 0.05 compared with IsoPC group

First, to validate the efficiency of RNA interference, the expression of Ubc9 expression was evaluated by Western blotting (Fig. 3a; *p* < 0.01). Next, the neuroblastoma cells were transfected with siRNA for 24 h prior to the exposure of isoflurane or treatment of OGD injury. The cell viability was assessed 24 h after OGD using the MTT assay. For neuroblastoma cells only subjected to 3-h OGD, cell viability of Ubc9 siRNA-transfected cells was lower than that of non-transfected cells (*p* < 0.05); however, there was no statistical difference between Ubc9-overexpressed cells and non-transfected cells. When subjected to isoflurane preconditioning plus OGD injury, Ubc9 lentivirus-transfected cells showed a 6.47 % increase in cell viability compared to non-transfected cells (*p* < 0.05). However, Ubc9 siRNA-transfected cells showed a 9.86 % decrease in cell viability (*p* < 0.05). The results showed that a knockdown of Ubc9 can attenuate the neuroprotective effect of isoflurane preconditioning and upregulation of Ubc9 was effective in improving survival of cells.

### Isoflurane Preconditioning Improved Neurologic Scores and Reduced Infarct Volume in Rats with MCAO

**Fig. 4:**
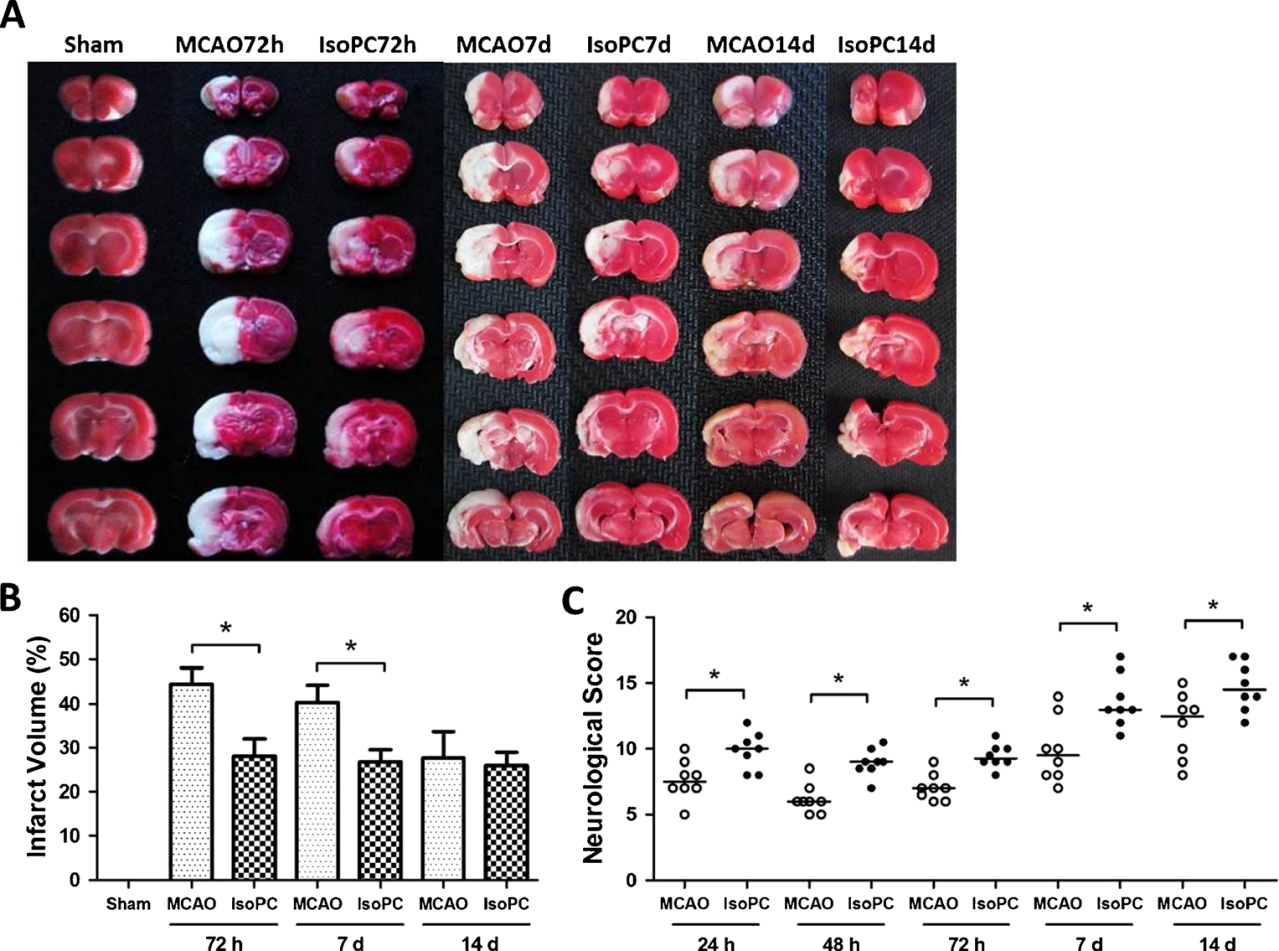
**a** Representative photograph showing the examples of sizes of infarct volume in different groups. Brain slices stained with 2,3,5-triphenyltetrazolium chloride (TTC) were from three groups: sham, MCAO, and IsoPC (isoflurane preconditioning plus MCAO), respectively. **b** Histograms showing the comparison of percentages of infarct volume among the three groups at the three corresponding time points (*n* = 8 for each group). Data are presented as mean ± SEM. *p < 0.05 compared with the MCAO group. **c** Comparisons of neurological scores among sham, MCAO, and IsoPC (isoflurane preconditioning plus MCAO) groups at each time point. Each *circle* represents the score for a single rat (*n* = 8 for each group). The *horizontal bar* indicates the median value of each group. *p < 0.05 compared with the MCAO group

Changes in physiologic parameters during isoflurane preconditioning and controlled oxygen inhalation are shown in Table 1. There were no statistical differences between the isoflurane inhalation and oxygen inhalation groups among the corresponding time points. Rats preconditioned with 2 % isoflurane 1 h/day for 5 days had significantly smaller brain infarct volumes than those without isoflurane precondionintg at 72 h and 7 days after the MCAO (*p* < 0.05). There were no significant differences between the MCAO 14-day and IsoPC 14-day groups (Fig. 4a). Consistent with the neuropathological data, isoflurane preconditioning also improved the neurological manifestation at 24, 48, 72 h, 7, and 14 days after MCAO (*p* < 0.05; Fig. 4c).

**Table 1:** Data of arterial gas anyalysis

Group	Before ischemia		During ischemia		After ischemia	
	MCAO	Pre + MCAO	MCAO	Pre + MCAO	MCAO	Pre + MCAO
pH	7.44 ± 0.02	7.45 ± 0.01	7.46 ± 0.01	7.45 ± 0.03	7.44 ± 0.01	7.46 ± 0.01
PaO_2_ (mmHg)	136 ± 5.6	134 ± 4.5	133 ± 5.1	135 ± 5.2	132 ± 5.5	134 ± 4.8
PaCO_2_ (mmHg)	38 ± 1.5	36 ± 1.2	39 ± 1.4	36 ± 1.1	35 ± 1.5	37 ± 1.3

Isoflurane preconditioning and oxygen inhalation data for arterial gas was obtained before, during, and after 10 min of preconditioning, using a separate group of rats. There was no significant difference between the two groups at the corresponding time points (*n* = 5 for each group). Data are presented as mean ± SEM

*MCAO* middle cerebral artery occlusion

### Isoflurane Preconditioning Reversed the Decline of Ubc9 Expression Induced by MCAO

**Fig. 5:**
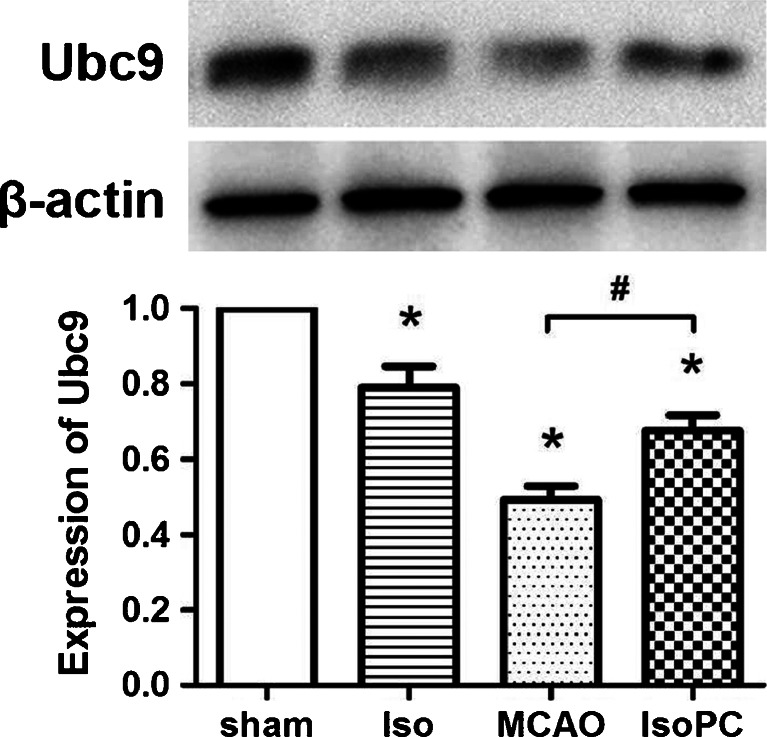
Western blot analysis for relative protein expression rate of Ubc9/β-actin in Sham, Iso, MCAO, and IsoPC (isoflurane preconditioning plus MCAO) groups 4 h after reperfusion. The *upper part* is the photo of representative Ubc9 and corresponding β-actin blotting bands. The *lower part* is the histograms showing the results of densitometric analysis (*n* = 6 for each group). Data are presented as mean ± SEM. *p < 0.05 compared with the sham group. #*p* < 0.05 compared with the MCAO group

The expression level of Ubc9 in the brain significantly decreased 4 h after MCAO surgery, compared with the sham group. Isoflurane preconditioning significantly reversed this decrease (*p* < 0.05; Fig. 5).

### Downregulation of Ubc9 Attenuated Isoflurane-Induced Neuroprotection Against Ischemia-Reperfusion Injury

**Fig. 6:**
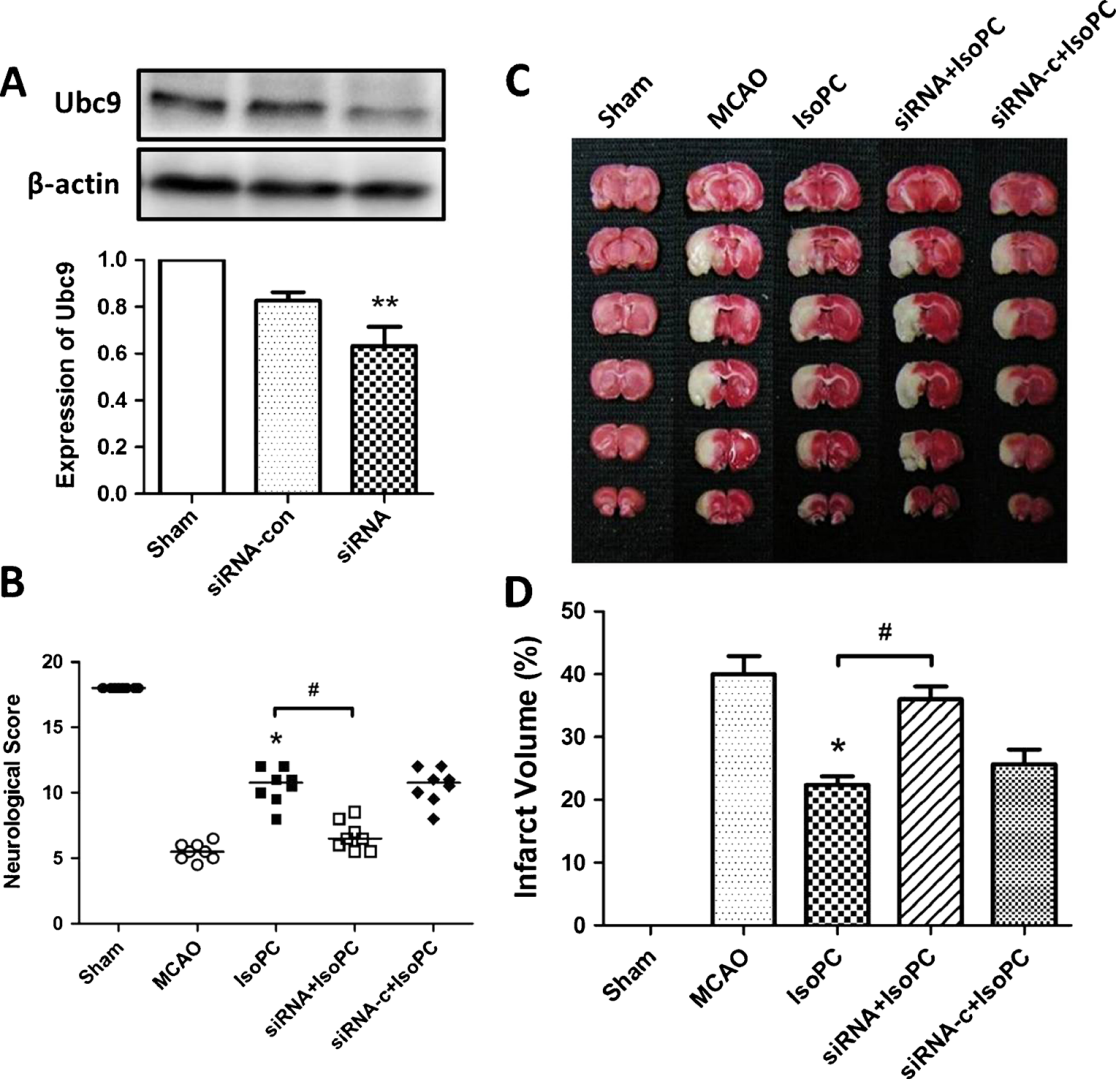
**a** Western blot analysis showed the relative expression rate of Ubc9/β-actin in rats. The rats were divided into three groups: sham, untreated rats; siRNA, rats injected with Ubc9 siRNA; siRNA-c, rats treated with Allstars Negative Control siRNA. Data are presented as mean ± SEM. **p < 0.01 vs. sham group. **b** The effect of Ubc9 siRNA on neurobehavioral manifestations. Each *circle* represents the score for a single rat. The *horizontal bar* indicates the mean value for each group. *p < 0.05 vs. MCAO group. #*p* < 0.05 vs. IsoPC group. **c** Comparisons of percentages of infarct volume among the five groups. *p < .05 vs. MCAO group. #*p* < 0.05 vs. IsoPC group

To verify the efficiency of siRNA in vivo, Ubc9 siRNA or nonsense siRNA was stereotaxically injected into the lateral ventricle. At 48 h after injection, the rat brain was harvested to measure the efficiency of siRNA using Western blotting. We found that Ubc9 siRNA significantly reduced the expression of Ubc9 protein (*p* < 0.01), but nonsense siRNA had no such effect (Fig. 6a).

At 24 h after intracerebroventricular microinjection of Ubc9 siRNA, the animals were subjected to subsequent treatments 24 h after reperfusion. Ubc9 siRNA (siRNA + IsoPC group) showed increased cerebral infarct volumes compared with those microinjected with nonsense siRNA (siRNA-c + IsoPC group) (Fig. 6c; *p* < 0.05). Ubc9 siRNA attenuated the improvement of the neurobehavioral manifestation induced by isoflurane preconditioning at the time points examined, as shown by the neurologic scores (Fig. 6d). Also, there were no detectable differences between the siRNA + IsoPC and MCAO groups in infarct volumes (*p* > 0.05), indicating that Ubc9 has an active role in the anti-ischemia-reperfusion injury effect of isoflurane preconditioning.

## Discussion

The aim of the present study was to test the hypothesis that the SUMO E2 conjugation enzyme, Ubc9, is involved in the neuroprotective effect of isoflurane preconditioning against cerebral ischemic injury. We discovered that ischemic injury significantly decreased the level of Ubc9 protein and isoflurane preconditioning can reverse this decrease. The ischemic tolerance induced by isoflurane preconditioning is diminished by the downregulation of Ubc9 via RNA interference. This indicates that changes in the pattern of protein modification by SUMO E2 conjugation enzyme Ubc9 are involved in the induction of the neuroprotective effect by isoflurane preconditioning.

Despite the benefit of ischemic preconditioning on attenuating the ischemic injury, its application is still invasive and impractical in brain ischemia for clinical practice. Therefore, many non-ischemic preconditioning maneuvers have recently been investigated in vitro and in vivo. Our previous studies have shown that preconditioning with hyperbaric oxygen and volatile anesthetic agents, such as sevoflurane and isoflurane, can induce ischemic tolerance in cerebral [25, 26] and spinal cord ischemia [4]. Since the volatile anesthetics have been widely used in surgery procedures, volatile anesthetic preconditioning has the potential to be a practicable method for preventing perioperative ischemic reperfusion injury. The mechanism of isoflurane preconditioning involves anti-oxidative pathways and K-ATP and TREK-1 channel activation [27, 28]. However, the underlying mechanism, especially the protein modulation after isoflurane preconditioning, remains unclear.

Evidence suggests that the ubiquitin proteasome system and SUMOylation are important for neuroprotective stress responses under conditions of transient cerebral ischemia. Our previous study found that inhibition of ubiquitin-conjugated protein aggregation is involved in the formation of cerebral ischemic tolerance by isoflurane preconditioning [21]. However, whether SUMOylation is involved and the precise targets of SUMO conjugation in ischemic tolerance induced by isoflurane preconditioning have not been previously identified.

The SUMO conjugation pathway relies on enzymes, such as E1 and E2. Since Ubc9 is believed to be the only SUMO-conjugating enzyme, it is an important target to activate or block SUMO conjugation. Overexpressing or downregulation of Ubc9 has been used to explore the possible role of SUMOylation under certain experimental conditions [29–31]. In the present study, we showed that downregulation of Ubc9 via RNA interference can reverse the neuroprotective effect of isoflurane preconditioning. Our results also indicated that isoflurane preconditioning significantly elevated Ubc9 expression in the early stage of reperfusion or reoxygenation in vivo and in vitro. These findings indicate that SUMO conjugation is involved in the neuroprotection of isoflurane preconditioning.

Recent findings have shown that a number of cell-signaling pathways may be the target of SUMO-dependent signaling processes and recovery following ischemic stroke, as well as showing a tightly controlled cross talk between the ubiquitin and SUMO conjugation systems [32]. Deep hypothermia which exhibits neuroprotection against ischemic injury induced the SUMO-conjugating enzyme Ubc9 nuclear translocation, indicating that the increase of SUMO-conjugated proteins level in the nucleus [33]. These results suggest that activation of SUMO conjugation through the increase and translocation of Ubc9 might help cells withstand the stress conditions caused by a transient period of hypoxia. However, available evidence did not clearly show whether Ubc9 was a cause or consequence of the neuroprotection.

Although Ubc9 is known to be the only E2 conjugase, there have been many reports identifying Ubc9 as a multifunctional protein that is independent of its role as a SUMOylation-conjugating enzyme. It can act as a regulator of nuclear transport, a transcriptional cofactor, or a helper for virus production [34]. SUMOylation broadly affects the biological network, and the process of SUMO conjugation is reversible and highly dynamic. Therefore, it is unclear how these complex processes are altered in the context of ischemia, preconditioning, and tolerance. Although mechanisms for OGD-regulated SUMOylation are still unknown, we can speculate that it will require posttranslational modification or differential expression of components of the SUMO conjugation machinery. Small ubiquitin-like modifier modification has been described to actively participate in NFκB regulation, and Liu et al. [35] documented that adenosine signaling resulted in significant accumulation of SUMOylation with subsequent attenuation of NFκB activation using models of hypoxia followed by reoxygenation. Other researchers have found that the internalization of GluK2 following SUMO modification activated the MLK3–JNK3 pathway, which may be responsible for ischemic neuronal cell death [36]. Moreover, protein SUMOylation has an important and previously unsuspected role in synaptic trafficking of AMPARs that underlies homeostatic scaling to alter synaptic transmission to compensate for changes in network activity [37]. Cimarosti et al. [38] investigated posttranslational modification by SUMO and discovered that downregulation of AMPARs and KARs may have important roles in the pathophysiological responses to ischemia in different animal stroke models. What remains unclear is whether SUMOylation of proteins following ischemia is part of a protective process or the cause of cell death.

In conclusion, the data from our present study show that preconditioning with isoflurane protects neurons from ischemic injury and this effect is partly mediated by Ubc9, suggesting that protein SUMOylation is involved in the cellular response to ischemia tolerance. However, in the absence of Ubc9 specifically activating or inhibiting SUMO conjugation, resolution of questions concerning the signaling properties of posttranslational modification will require further studies in transgenic animals.
